# Supplementary material for: Mimicking efferent nerves using a graphdiyne-based artificial synapse with multiple ion diffusion dynamics
Source: Nat Commun. 2021 Feb 16;12:1068. doi: 10.1038/s41467-021-21319-9 (PMC7886898; doi:10.1038/s41467-021-21319-9)
Supplement: Supplementary file 1 — Supplementary Information [file 41467_2021_21319_MOESM1_ESM.pdf]

Supplementary Information

Mimicking efferent nerves using a graphdiyne-based artificial synapse with multiple ion diffusion dynamics

Wei *et al.*

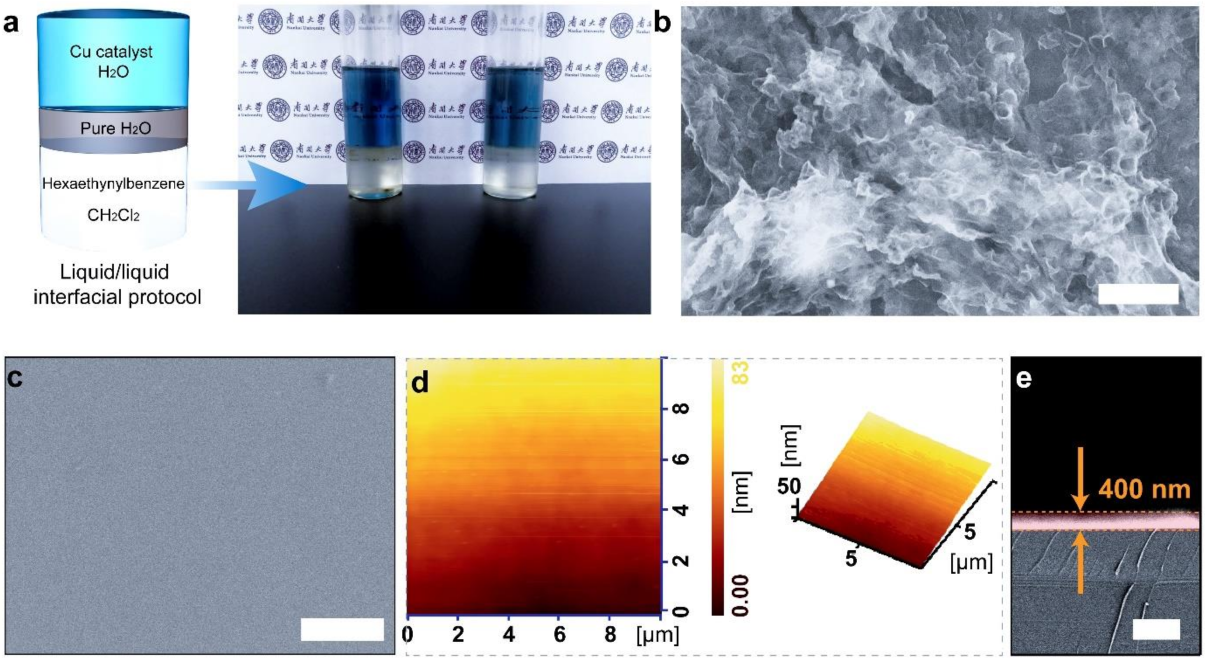

Supplementary Fig. 1 a Schematic illustration of the liquid/liquid interfacial protocol and the corresponding digital photo. GDY is obtained from the middle layer of the glass cylinder. The catalyst and the hexaethynylbenzene (HEB) monomer were placed above and below the intermediate layer of pure water. **b** SEM image of the obtained GDY (scale bar: 4 μm). **c** SEM image (scale bar: 400 nm), **d** AFM images, **e** cross-section SEM image (scale bar: 1 μm) of the spin-coated GDY film.

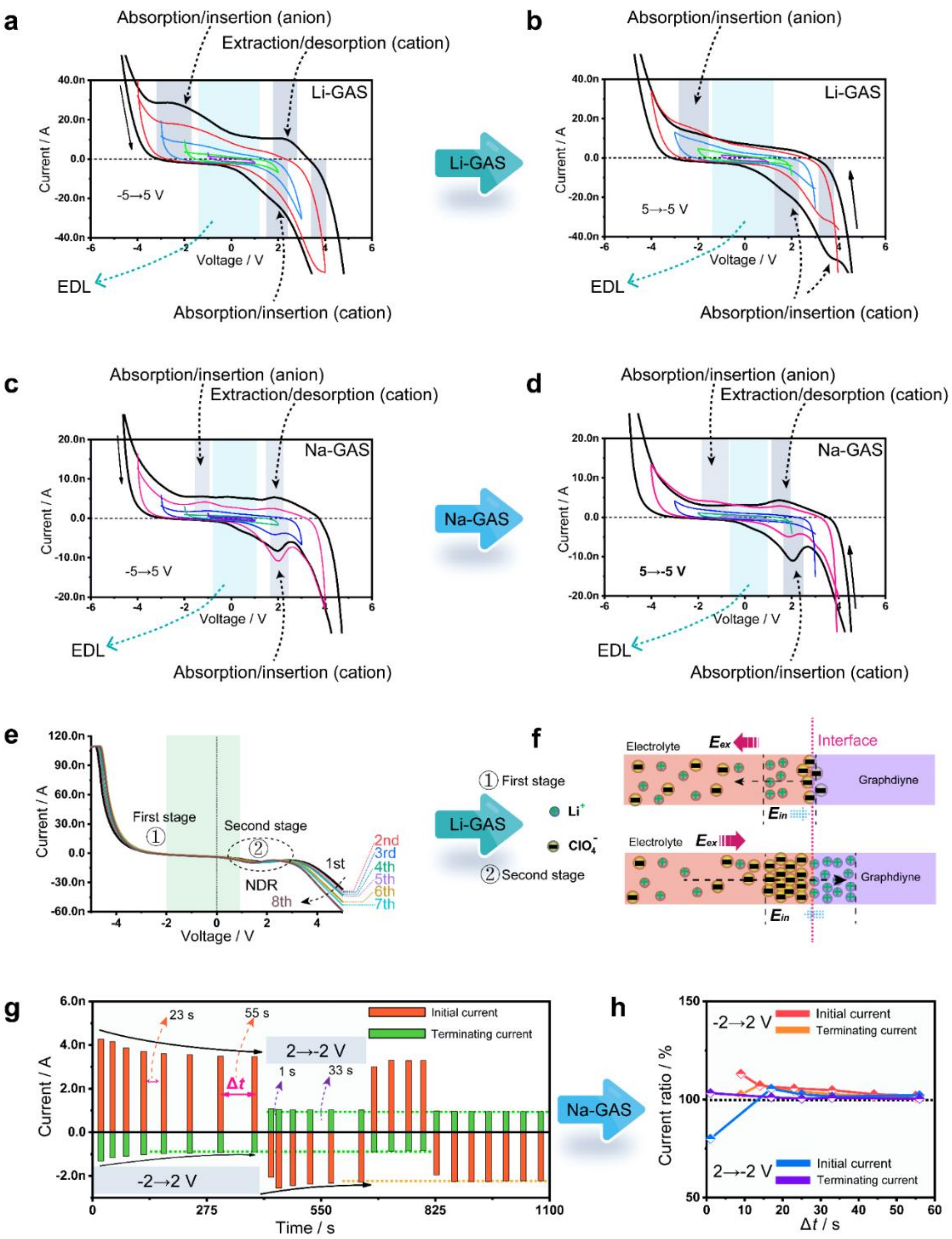

Supplementary Fig. 2 *I*–*V* curves measured in sweep cycles of **a**, **c** -5 to 5 V, …, -1 to 1 V, and **b**, **d** 5 to -5 V, …, 1 to -1 V in Li-GAS (**a** and **b**) and Na-GAS (**c** and **d**), respectively. The curve shows the ion dynamics process, electric double layer process, and electrochemical doping process of the device under the action of the pulse. **e** *I*–*V* curves and **f** schematic illustration of ions' dynamic diffusion in a sweep cycle of -5 → 5 V. Eight sweeping cycles were applied in Li-GAS. **g** Initial and termination of current values for Na-GAS in different voltage linear sweep modes. **h** Initial and termination current ratio (I_{pre}/I_{post}) versus time interval ($\Delta t_{post\text{-}pre}$) between successive pulses in Na-GAS.

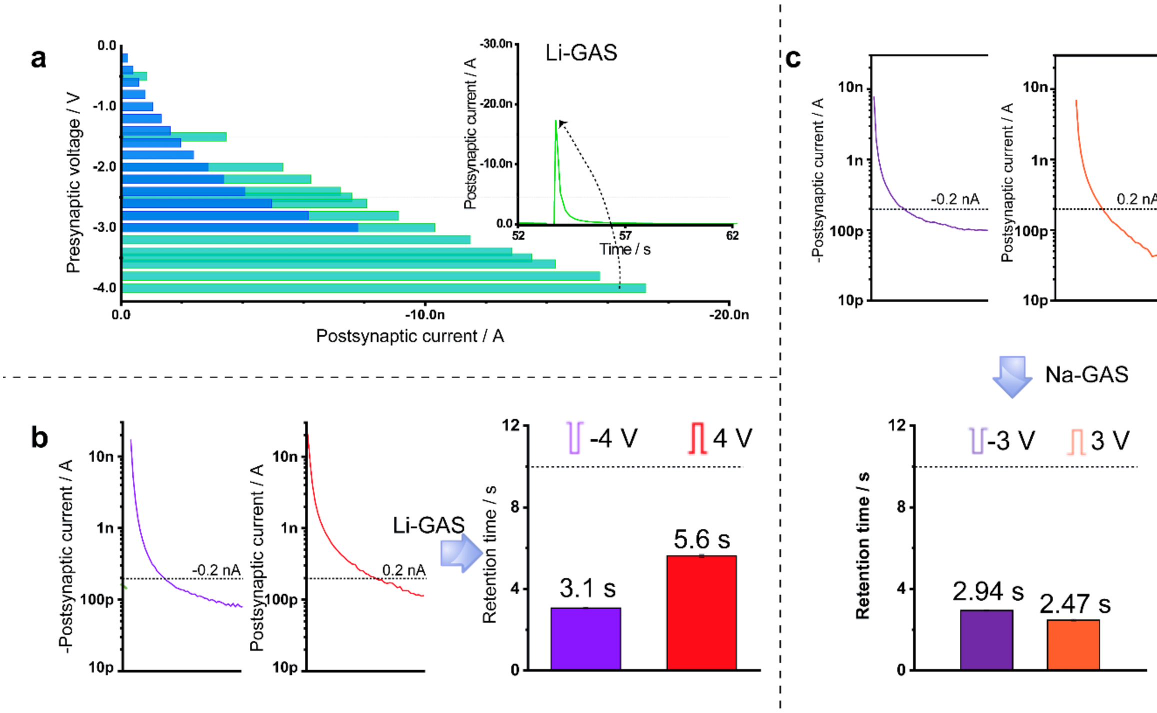

Supplementary Fig. 3 **a** Peak value of postsynaptic current with different negative pulse amplitudes in Li-GAS (green) and Na-GAS (blue). Inset: Postsynaptic current triggered by a single spike in Li-GAS. Retention curve under positive and negative pulses and corresponding retention time in **b** Li-GAS and **c** Na-GAS. To further demonstrate the short-term plasticity of the device, the retention curve was analyzed and the current decayed to near the baseline within a few seconds (< 6 s).

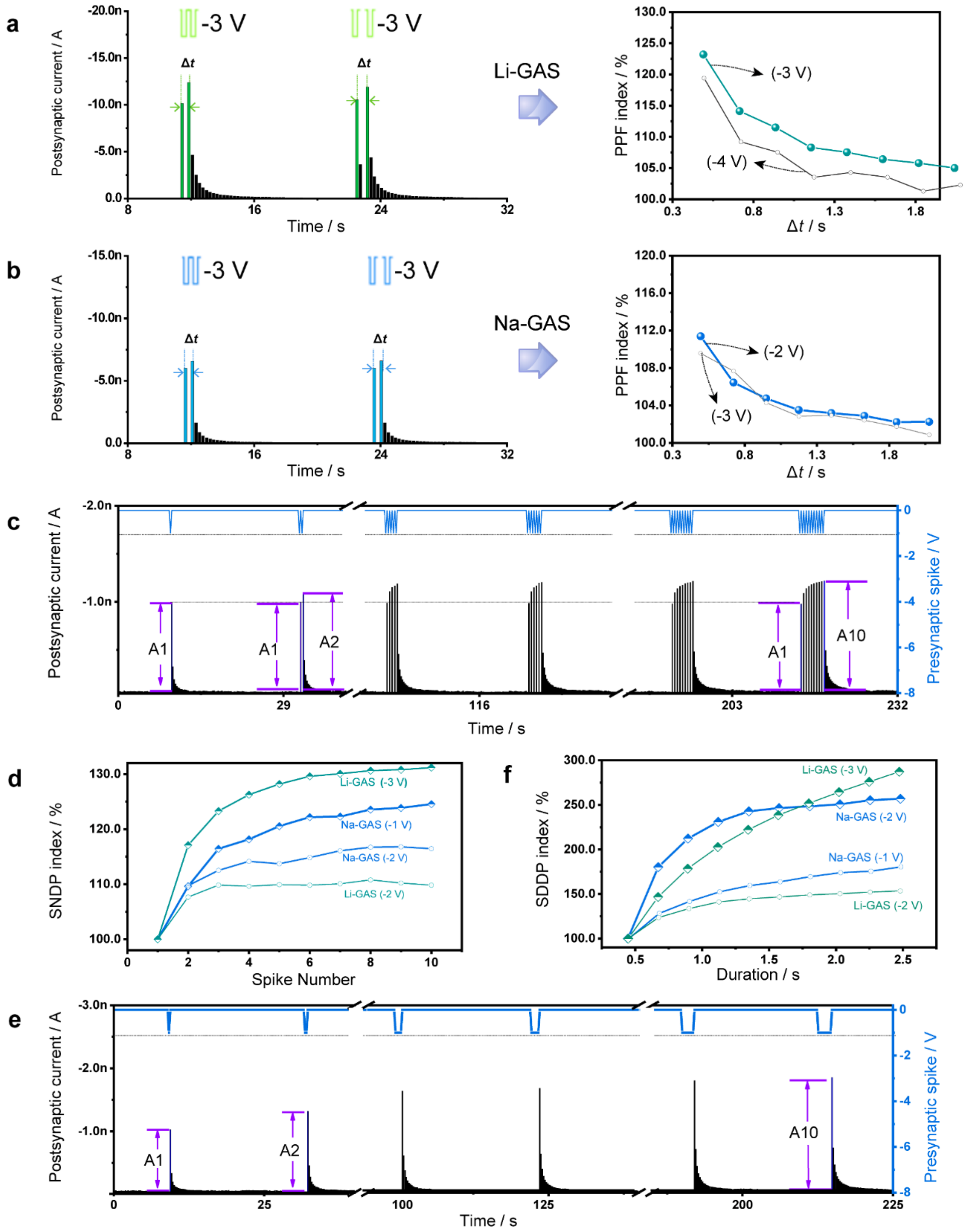

Supplementary Fig. 4 Postsynaptic currents triggered by two consecutively negative pulses and corresponding PPF indexes in **a** Li-GAS and **b** Na-GAS. **c** Postsynaptic current at different pulse number in Na-GAS. **d** Gain of postsynaptic currents (SNDP index; A10/A1×100%) plotted as a function of presynaptic pulse numbers in Li-GAS and Na-GAS. **e** Postsynaptic current at different pulse duration in Na-GAS. **f** Gain of postsynaptic currents (SDDP index; A10/A1×100%) plotted as a function of presynaptic pulse duration in Li-GAS and Na-GAS.

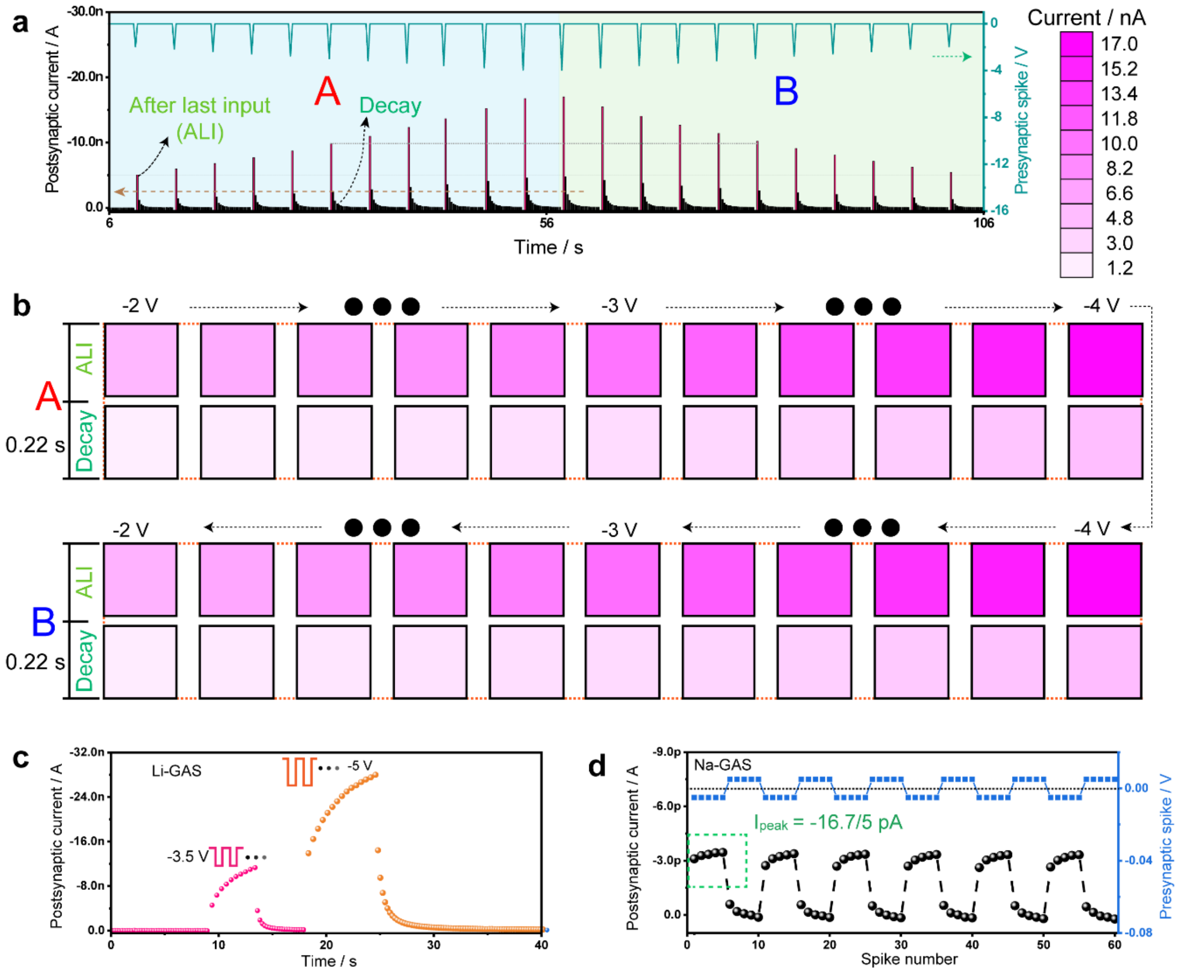

Supplementary Fig. 5 **a** Postsynaptic current triggered by nonidentical negative pulse sequence and **b** the corresponding current (peak current and attenuation current) color image in Li-GAS. **c** Postsynaptic current triggered by negative pulses with amplitudes of -3.5 and -5 V in Li-GAS. **d** Postsynaptic current triggered by 5 negative and 5 positive pulses with an amplitude of ± 5 mV in Na-GAS. Here, the sum of the first five current peaks is -16.7 pA. The average value of the continuously enhanced current peak I_{peak} is 3.34 pA (-16.7/5 pA). Hence, the average power consumed by a single synaptic event is 16.7 fW (given by $I_{peak}\times$ V).

Supplementary Table 1. Comparison of pulse amplitude, power and energy consumption of artificial synapse devices.

Device structures	Pulse amplitude / mV	Power consumption / pW	Energy consumption / pJ	Reference
Ag-Cluster-Doped TiO_2<br>Memristor size: π × 50 × 50 μm^2	±1000	/	SET: 26.0<br>RESET: 22.9	1
IGZO/Alkylated Graphene Oxide<br>Channel length and width:<br>10 × 10 μm^2	-500	/	Capacitive: 136<br>Resistive: 14.3	2
$CH_3NH_3PbBr_3$ Single Crystalline<br>Channel length and width:<br>100 × 260 μm^2	-30	0.0157	0.0143	3
MoS_2/DEME-TFSI<br>Channel length and width: 9 × 20 μm^2	2000<br>50000	/	Ionotronic: 4.8<br>Electronic: 13000	4
WO_3/DEME-TFSI<br>Channel size: 500 × 50 μm^2	V_G: 600<br>V_{SD}: 300	519	36	5
PbS Quantum Dots/Ga_2O_3<br>Memristor size: π × 50 × 50 μm^2	SET: 120~260<br>RESET: -50~-190	SET: ~1000<br>RESET: ~1000000	/	6
$FAPbBr_3$<br>Device area: 0.1 mm^2	/	/	2300	7
Silicon Nanocrystals<br>Device area: 2 × 2 mm^2	/<br>(Optical Pulse)	/	0.7	8
In-Doped TiO_2<br>100 μm gap (source-drain)	/<br>(Optical Pulse)	/	2.41	9
IZO/Nanogranular SiO_2<br>Channel thickness: 20 nm	300<br>(Gate bias: -700)	/	15	10
PEDOT: PSS/Nafion/<br>PEDOT: PSS-PEI<br>Device area: 10^3 μm^2	/	/	~10	11
WO_3/Nafion-117 resin<br>Device area: 0.6 × 1.2 mm^2<br>Channel length and width:<br>100 × 500 μm^2	250	125000	625	12
P3HT: PEO/PS-PMMA-PS/<br>EMMI-TFSI<br>Channel length: 300 nm	Presynaptic spike: -1<br>(V_D: 20)	/	0.00123	13
WSe_2/PEO: $LiClO_4$<br>Source–drain distance: ≈1 μm	100	/	0.03	14
PETE-S/ETE-S: NaCl<br>Channel length and width:<br>30 × 1000 μm^2	V_G: 0.5<br>V_G: 20	/	1.1<br>1491	15
$NaSbS_2$<br>/	Constant bias: 1000	/	5.75	16
MXene/PEO: $LiClO_4$<br>/	80	6.4	~5.6	17
**GDY/PEO: $NaClO_4$**<br>**Device area: π/4 × 0.33 × 0.33 mm^2**	**5**	**0.0167**	**/**	**this work**

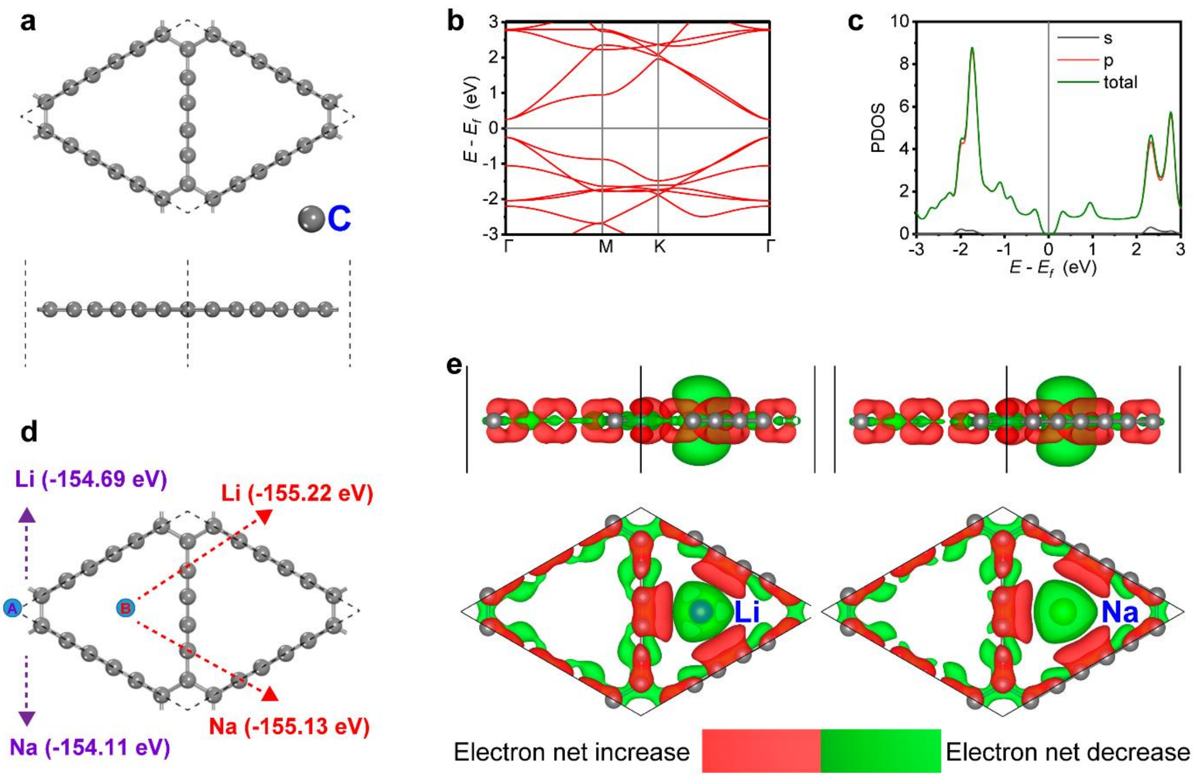

Supplementary Fig. 6 a Top- and side- viewed chemical structures of monolayer GDY. **b** Band structures of monolayer GDY. **c** PDOS of monolayer GDY. The Fermi level is set to zero. **d** Schematic diagram of the two possible locations for one Li or one Na atom. **e** Top- and side-viewed figures of difference charge density for Li and Na adsorbed GDY. Red and green colors indicate electron net increase and decrease, respectively.

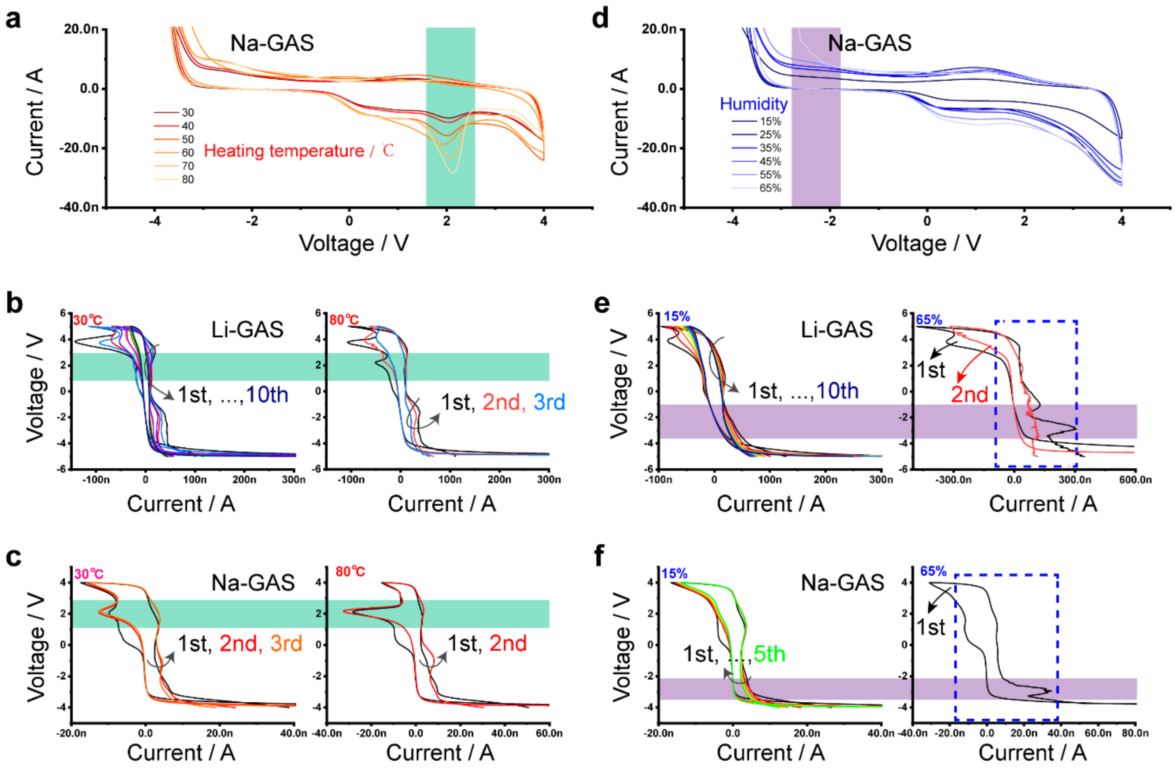

Supplementary Fig. 7 *I*–*V* curves of Na-GAS in a sweep cycle of -4 to 4 V measured **a** after heat (for ten minutes at each temperature from 30 to 80 °C) and **d** humidity (for an hour at each relative humidity from 15% to 65%) treatments. The current changes of **b** Li-GAS and **c** Na-GAS under different cycles at 30 and 80 °C, respectively. The current changes of **e** Li-GAS and **f** Na-GAS under different cycles at relative humidities of 15% and 65%, respectively.

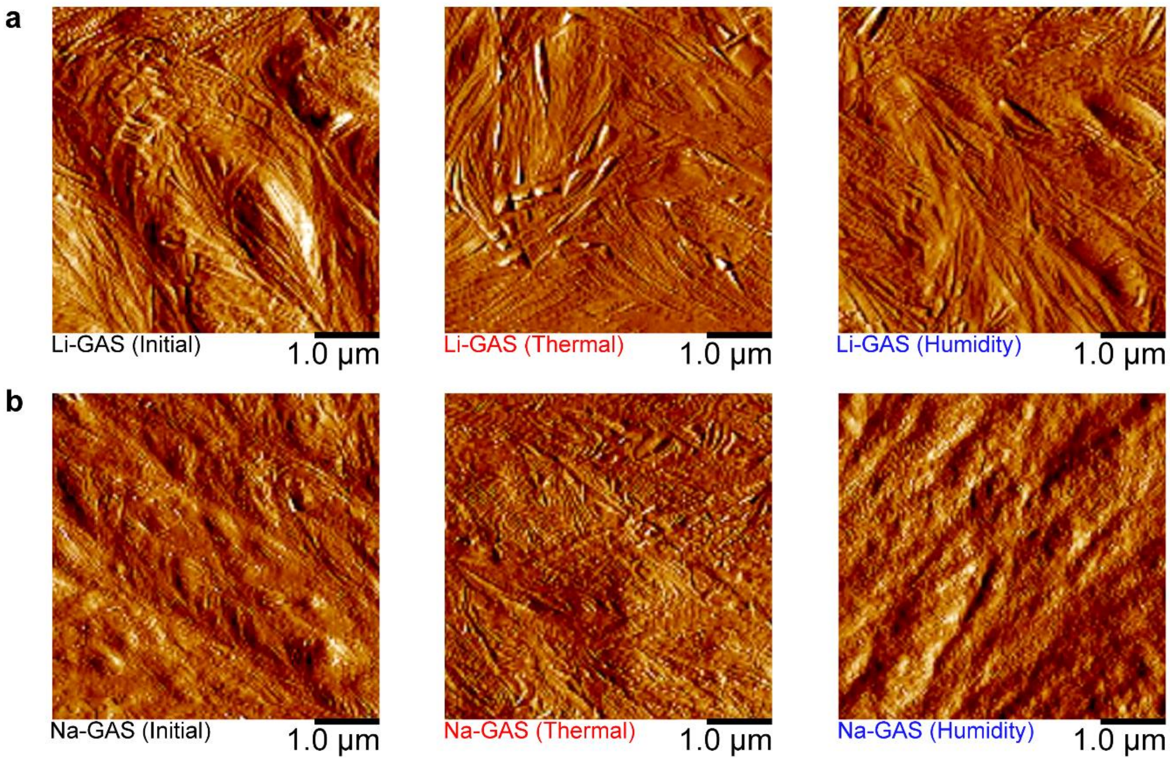

Supplementary Fig. 8 The surface morphology of the electrolytes in **a** Li-GAS and **b** Na-GAS in the initial state and after heating or environmental humidity treatments.

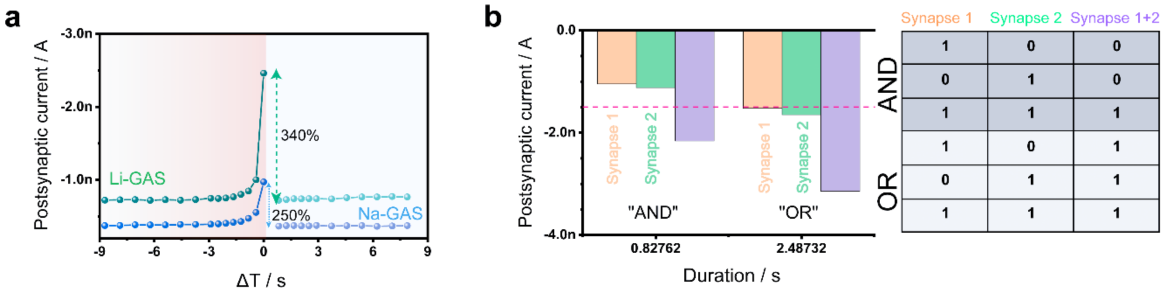

Supplementary Fig. 9 a The amplitude of postsynaptic current at T = 0 plotted as a function of Δ*T*. **b** A spiking logic response by two presynaptic inputs (synapse 1, synapse 2, synapse 1 + synapse 2) with different pulse duration and the correspomding truth tables for "AND" logic (duration: 0.82762 s) and "OR" logic (duration: 2.48732 s), respectively.

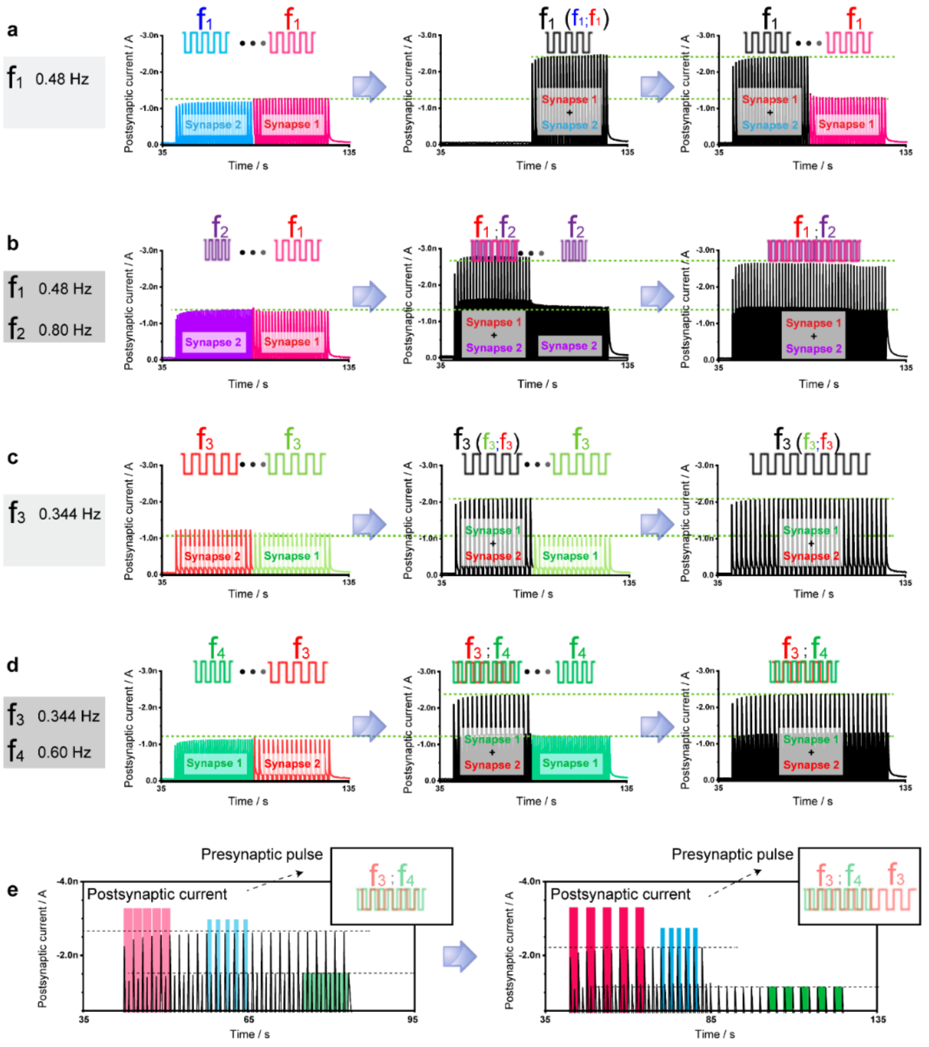

Supplementary Fig. 10 **a** Postsynaptic current triggered by two presynaptic inputs at 0.48 Hz. **b** Postsynaptic current triggered by two presynaptic inputs with different frequencies (0.48 and 0.8 Hz). **c** Postsynaptic current triggered by two presynaptic inputs at 0.344 Hz. **d** Postsynaptic current triggered by two presynaptic inputs with different frequencies (0.344 and 0.60 Hz). **e** The current peak shape of the postsynaptic current, used to estimate the frequency and amplitude of the presynaptic pulse. The response triggered by four low-frequency (0.344, 0.4, 0.6, and 0.8 Hz) presynaptic pulses can be well integrated and output. Analysis of the temporal profiles of postsynaptic currents in these four cases demonstrates the possibility of a bioinspired approach to the identification of the frequency of presynaptic pulse sequences, which can be estimated from the shape of postsynaptic signals and peak-valley time interval. Furthermore, GAS can identify the frequency of presynaptic inputs to a certain extent based on the postsynaptic current to infer and analyze the sensory information transmitted from afferent nerves.

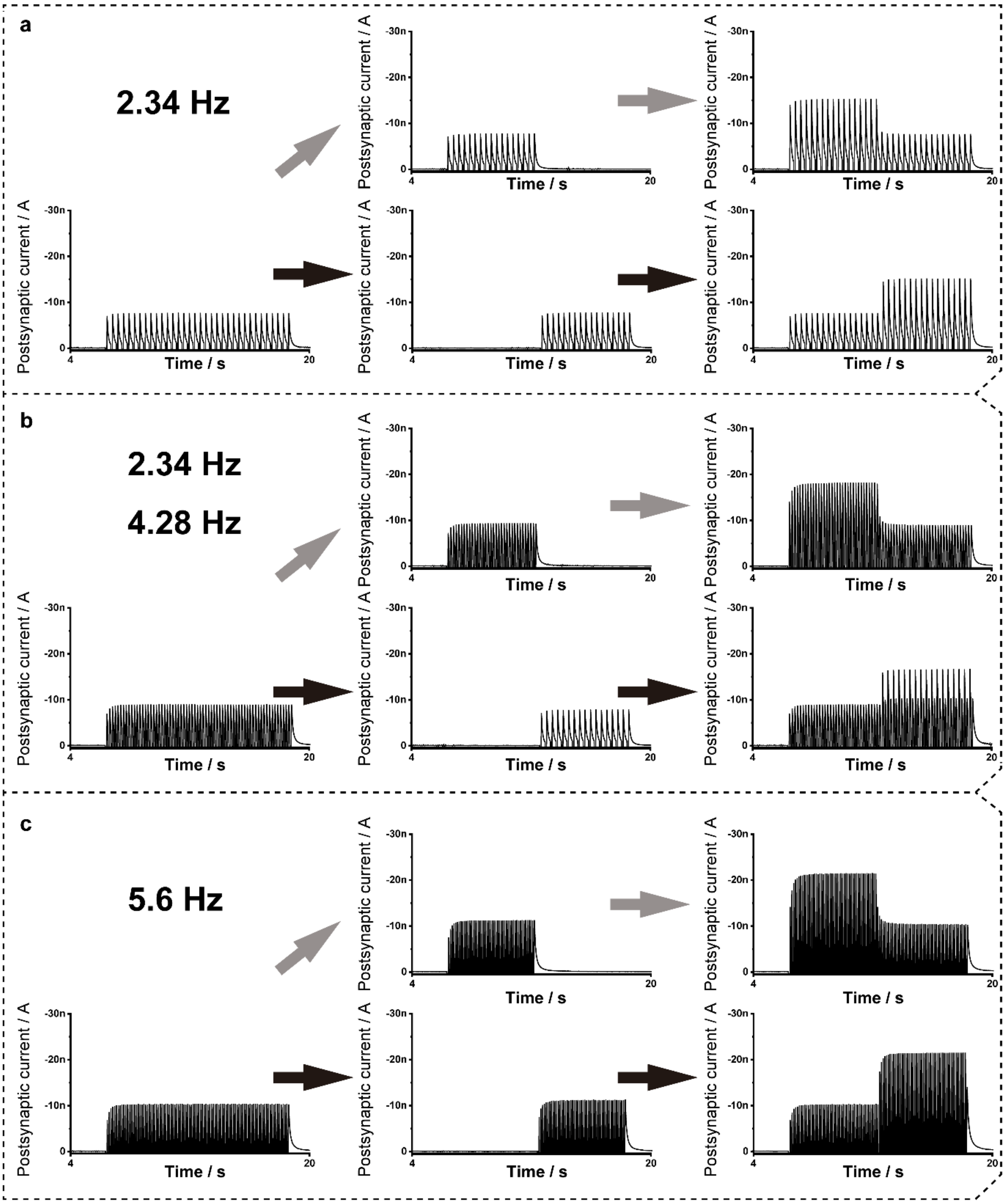

Supplementary Fig. 11 a Postsynaptic current triggered by one or two presynaptic inputs at 2.34 Hz. **b** Postsynaptic current triggered by one or two presynaptic inputs with different frequencies (2.34 and 4.28 Hz). **c** Postsynaptic current triggered by one or two presynaptic inputs at 5.6 Hz. Here, as the pulse frequency continues to increase, the synapse output terminal is still sensitive to the pulse timing of the two input terminals. That is, when the applied two sets of pulses overlap in time (Supplementary Fig. 11a and c), the synapse weight will double. If these two sets of pulse frequencies are different, this gain effect can still be observed (Supplementary Fig. 11b).

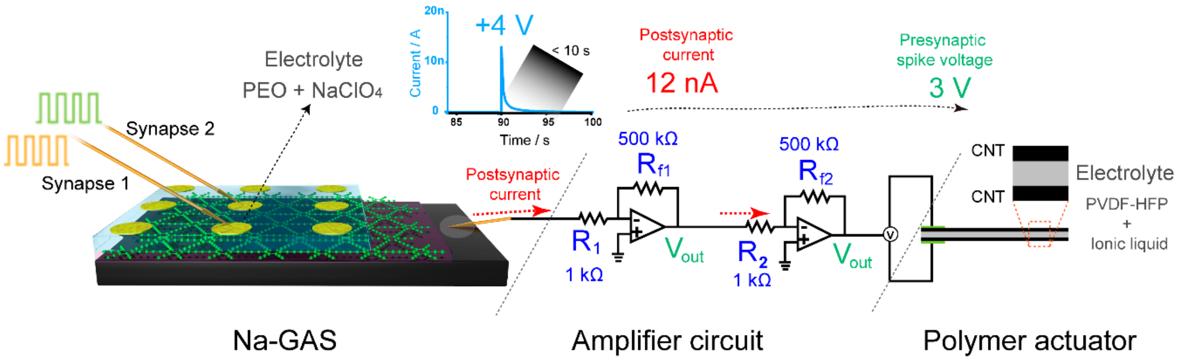

Supplementary Fig. 12 Diagram of synaptic the device- amplifier circuit-polymer actuator system. As a demonstration, the nano-amp-level postsynaptic current of a single device (Na-GAS) is amplified and the motor neuron synaptic potential is output to drive the artificial muscle (polymer actuator). Here, an operational amplifier is introduced to output the desired voltage and operate the actuator. The amplifier circuit amplifies the input voltage (+4 V) by 250,000 times to reach the working voltage of the actuator (~ 3 V). The bottom electrode of the synaptic device is connected to the amplifier circuit to convert currents (~12 nA) to output voltages, so that the actuator can be operated. One end of the bottom electrode of the synaptic device is coated with silver paste and dried in air. Copper wires are used for connection.

References

1 Yan, X. *et al.* Memristor with Ag-Cluster-Doped TiO_2 Films as Artificial Synapse for Neuroinspired Computing. *Adv. Funct. Mater.* **28**, 1705320 (2018).

2 Sun, J. *et al.* Optoelectronic Synapse Based on IGZO-Alkylated Graphene Oxide Hybrid Structure. *Adv. Funct. Mater.* **28**, 1804397 (2018).

3 Gong, J. *et al.* Lateral Artificial Synapses on Hybrid Perovskite Platelets with Modulated Neuroplasticity. *Adv. Funct. Mater.* **30**, 2005413 (2020).

4 John, R. A. *et al.* Synergistic Gating of Electro-Iono-Photoactive 2D Chalcogenide Neuristors: Coexistence of Hebbian and Homeostatic Synaptic Metaplasticity. *Adv. Mater.* **30**, 1800220 (2018).

5 Yang, J. T. *et al.* Artificial Synapses Emulated by an Electrolyte-Gated Tungsten-Oxide Transistor. *Adv. Mater*. **30**, e1801548 (2018).

6 Yan, X. *et al.* Self-Assembled Networked PbS Distribution Quantum Dots for Resistive Switching and Artificial Synapse Performance Boost of Memristors. *Adv. Mater.* **31**, 1805284 (2019).

7 John, R. A. *et al.* Ionotronic Halide Perovskite Drift-Diffusive Synapses for Low-Power Neuromorphic Computation. *Adv. Mater.* **30**, e1805454 (2018).

8 Tan, H. *et al.* Broadband optoelectronic synaptic devices based on silicon nanocrystals for neuromorphic computing. *Nano Energy* **52**, 422-430 (2018).

9 Karbalaei Akbari, M. & Zhuiykov, S. A bioinspired optoelectronically engineered artificial neurorobotics device with sensorimotor functionalities. *Nat. Commun.* **10**, 3873 (2019).

10 Zhu, L. Q., Wan, C. J., Guo, L. Q., Shi, Y. & Wan, Q. Artificial synapse network on inorganic proton conductor for neuromorphic systems. *Nat. Commun.* **5**, 3158 (2014).

11 van de Burgt, Y. *et al.* A non-volatile organic electrochemical device as a low-voltage artificial synapse for neuromorphic computing. *Nat. Mater.* **16**, 414-418 (2017).

12 Yao, X. *et al.* Protonic solid-state electrochemical synapse for physical neural networks. *Nat. Commun.* **11**, 3134 (2020).

13 Xu, W., Min, S.-Y., Hwang, H. & Lee, T.-W. Organic core-sheath nanowire artificial synapses with femtojoule energy consumption. *Sci. Adv.* **2**, e1501326 (2016).

14 Zhu, J. *et al.* Ion Gated Synaptic Transistors Based on 2D van der Waals Crystals with Tunable Diffusive Dynamics. *Adv. Mater.* **30**, e1800195 (2018).

15 Gerasimov, J. Y. *et al.* An Evolvable Organic Electrochemical Transistor for Neuromorphic Applications. *Adv. Sci.* **6**, 1801339 (2019).

16 Harikesh, P. C. *et al.* Cubic $NaSbS_2$ as an Ionic–Electronic Coupled Semiconductor for Switchable Photovoltaic and Neuromorphic Device Applications. *Adv. Mater.* **32**, 1906976 (2020).

17 Wei, H. *et al.* Redox MXene Artificial Synapse with Bidirectional Plasticity and Hypersensitive Responsibility. *Adv. Funct. Mater.* **31**, 2007232 (2020).
